# Management of cavernous sinus meningiomas: Consensus statement on behalf of the EANS skull base section

**DOI:** 10.1016/j.bas.2022.100864

**Published:** 2022-01-21

**Authors:** Marco V. Corniola, Pierre-Hugues Roche, Michaël Bruneau, Luigi M. Cavallo, Roy T. Daniel, Mahmoud Messerer, Sebastien Froelich, Paul A. Gardner, Fred Gentili, Takeshi Kawase, Dimitrios Paraskevopoulos, Jean Régis, Henry W.S. Schroeder, Theodore H. Schwartz, Marc Sindou, Jan F. Cornelius, Marcos Tatagiba, Torstein R. Meling

**Affiliations:** a Department of Neurosurgery, Centre Hospitalier Universitaire de Rennes/Pontchaillou, Rennes, France; b Faculty of Medicine, University of Rennes, Rennes, France; c MediCIS Research Group, INSERM UR1, UMR 1099 LTSI, France; d Faculty of Medicine, University of Geneva, Geneva, Switzerland; e Service de Neurochirurgie, Aix-Marseille Université, Assistance Publique-Hôpitaux de Marseille, Hôpital Nord, Marseille, France; f Department of Neurosurgery, Universitair Ziekenhuis Brussel, Vrije Universiteit Brussel, Laarbeeklaan, Brussels, Belgium; g Department of Neurological Sciences, Division of Neurosurgery, Università Degli Studi di Napoli Federico II, Naples, Italy; h Department of Neurosurgery, Centre Hospitalier Universitaire Vaudois, Lausanne, Switzerland; i Service de Neurochirurgie, Hôpital Lariboisière, Assistance Publique-Hôpitaux de Paris, APHP Nord, Paris, France; j Department of Neurological Surgery, University of Pittsburgh, Pittsburgh, PA, USA; k Department of Neurosurgery, Toronto Western Hospital, University Health Network, Toronto, Ontario, Canada; l Department of Neurosurgery, School of Medicine, Keio University, Tokyo, Japan; m Department of Neurosurgery, Barts Health NHS Trust, St. Bartholomew's and the Royal London Hospital, London, United Kingdom; n Department of Functional and Stereotactic Neurosurgery and Radiosurgery, Timone University Hospital, Marseille, France; o Department of Neurosurgery, Ernst Moritz Arndt University, Greifswald, Germany; p Department of Neurosurgery, Weill Cornell Medicine, New York Presbyterian Hospital, New York, USA; q University of Lyon, France; r IRR Flavigny, UGECAM Nord-Est, Nancy, France; s Pediatric Neurosurgery Department, Centre Hospitalier Régional de Nancy, Nancy, France; t Department of Neurosurgery, Heinrich Heine Universität, Düsseldorf, Germany; u Department of Neurosurgery, Uniklinik Tübingen, Tübingen, Germany; v Department of Neurosurgery, Geneva University Hospitals, Geneva, Switzerland

**Keywords:** Neurosurgery, Cavernous sinus, Meningioma, Microsurgery, Gross total resection, Cranial nerves, Radiosurgery, Radiotherapy, Pituitary, Consensus statement, Intracranial meningiomas, Gammaknife

## Abstract

**Introduction:**

The evolution of cavernous sinus meningiomas (CSMs) might be unpredictable and the efficacy of their treatments is challenging due to their indolent evolution, variations and fluctuations of symptoms, heterogeneity of classifications and lack of randomized controlled trials. Here, a dedicated task force provides a consensus statement on the overall management of CSMs.

**Research question:**

To determine the best overall management of CSMs, depending on their clinical presentation, size, and evolution as well as patient characteristics.

**Material and methods:**

Using the PRISMA 2020 guidelines, we included literature from January 2000 to December 2020. A total of 400 abstracts and 77 titles were kept for full-paper screening.

**Results:**

The task force formulated 8 recommendations (Level C evidence). CSMs should be managed by a highly specialized multidisciplinary team. The initial evaluation of patients includes clinical, ophthalmological, endocrinological and radiological assessment. Treatment of CSM should involve experienced skull-base neurosurgeons or neuro-radiosurgeons, radiation oncologists, radiologists, ophthalmologists, and endocrinologists.

**Discussion and conclusion:**

Radiosurgery is preferred as first-line treatment in small, enclosed, pauci-symptomatic lesions/in elderly patients, while large CSMs not amenable to resection or WHO grade II-III are candidates for radiotherapy. Microsurgery is an option in aggressive/rapidly progressing lesions in young patients presenting with oculomotor/visual/endocrinological impairment. Whenever surgery is offered, open cranial approaches are the current standard. There is limited experience reported about endoscopic endonasal approach for CSMs and the main indication is decompression of the cavernous sinus to improve symptoms. Whenever surgery is indicated, the current trend is to offer decompression followed by radiosurgery.

## Highlights

• A thorough evaluation of cavernous sinus meningiomas by a multidisciplinary team is mandatory.
• Microsurgery should be considered for aggressive lesions in young patients.
• Extended endoscopic approaches can be effective when combined with radiotherapy.
• Stereotaxic radiotherapy and stereotaxic radiosurgery offer excellent tumour control in small/asymptomatic lesions .

## 1 Introduction

Cavernous sinus meningiomas (CSMs) are the most common primary cavernous sinus tumours, occurring in circa 0.5 per 100′000 persons in the general population (Radhakrishnan et al., 1995). Yet, they represent only ∼1% of all intracranial meningiomas (Meling et al., 2019). CSMs constitute a specific subset of intracranial meningiomas, being mostly World Health Organization (WHO) grade I lesions with a meningothelial histology (Maiuri et al., 2019). Their clinical presentation, inherent to their specific location, often involves visual impairment, oculomotor perturbations and facial sensory changes. Endocrine dysfunction requiring long-term hormonal substitution may also occur.

The cavernous sinus (CS) has average dimensions of 2 cm long and 1 cm wide (Standring, 2008); it consists in a complex venous channel located in the parasellar space, limited by the inner (periosteal) dural fold and outer (meningeal) dural fold (Taptas, 1982, 1990; Umansky and Nathan, 1982; Umansky et al., 1994; Kawase et al., 1996), draining blood from the ophthalmic veins (superior and inferior) and the spheno-orbital sinus to the petroclival venous plexus, the superior petrosal sinus (to the sigmoid sinus) and the inferior petrosal sinus (to the jugular bulb). The CS has varying connections with the deep facial veins through the pterygoid plexus, as well as with superficial sylvian veins, rendering the surgical management of CSMs even more challenging. The CS encircles the pituitary complex and contains cranial nerves (CNs) III, IV, V_1_, V_2_ and VI, the cavernous segment of the internal carotid artery (ICA) and the peri-carotid sympathetic plexus (Dolenc, 2003; Standring, 2008; Kehrli et al., 1995). The cranial nerves are III, IV and VI are surrounded by an arachnoid sheath and arachnoidal granulations from which intra-cavernous sinus meningiomas arise (Kehrli et al., 1995).

CSMs originate either from the cavernous sinus (CS) itself or invade it secondarily from adjacent locations, such as the petrous bone, the petro-clival region, the anterior clinoid process, or the sphenoid wing (Abdel-Aziz et al., 2004; Shrivastava et al., 2005). They can also be part of even more complex central skull base meningiomas that invade altogether these structures. Once considered inoperable due to the concentration of critical neurovascular structures in the parasellar area, CSMs still pose formidable surgical challenges, which may be extremely difficult, even for advanced skullbase neurosurgeons. Because some, if not all, CSMs, infiltrate the surrounding CNs and ICA: this is specifically why complete resection along with the complete preservation of the CN function is very rarely possible (Larson et al., 1995; Shaffrey et al., 1999; Kotapka et al., 1994) (Larson et al., 1995; Shaffrey et al., 1999; Kotapka et al., 1994).

The CS was first surgically approached by Parkinson, 1965, 1998 in 1965; the 1980-ies and 1990-ies saw a phase where CSMs underwent increasingly aggressive surgical resections, with some authors using high-flow extracranial-intracranial (EC-IC) bypass surgery to eventually achieve gross total resections (GTRs) and CS exenterations (George et al., 2003; Sekhar et al., 1987; Sekhar and Moller, 1986; Sen and Sekhar, 1992). The morbidity of such procedures, which often required adjuvant radiotherapy (RT) despite aggressive resections, became increasingly unacceptable and this period was followed by a more prudent approach where RT (stereotaxic radiosurgery (SRS), stereotaxic radiosurgery or fractionated stereotaxic radiotherapy (f-SRT)) – either as first-line or adjuvant therapies – were shown as valid alternatives to aggressive surgical management, offering significantly lower morbidity and satisfactory progression-free and overall survivals (PFS and OS) (Azar et al., 2017; Brell et al., 2006; Correa et al., 2014; dos Santos et al., 2011; Hafez et al., 2015; Haghighi et al., 2015; Hasegawa et al., 2007; Hung et al., 2019; Lee et al., 2002; Litre et al., 2009; Metellus et al., 2010; Metellus et al., 2005; Nicolato et al., 2002a; Nicolato et al., 2002b; Pamir et al., 2005; Pollock and Stafford, 2005; Pollock et al., 2013; Roche et al., 2000; Selch et al., 2004; Shin et al., 2001; Skeie et al., 2010; Slater et al., 2012; Spiegelmann et al., 2002; Zeiler et al., 2012).

The most recent developments in microsurgical techniques, trans-nasal endoscopic approaches, intra-operative neuromonitoring speak against the total renunciation of microsurgical management of CSMs and offers new treatment options (Kaspera et al., 2015; Lave et al., 2020; Montaser et al., 2017; Truong et al., 2018). Altogether, these technological advances allow for safer tumour resections, ever since these techniques were implemented and mastered by well-trained skull-base surgeons. Weighing the risks and benefits of maximal safe resection, based on the assumption that the extent of resection (EOR) is inversely related to the rate of recurrence and size of the radiation field, skull base neurosurgeons must still be able and ready to propose surgery to very selected patients.

In spite of their benign nature, the evolution of CSMs might be unpredictable, as the clinical symptoms do not always correlate with tumour size or growth rate (Amelot et al., 2018). Since they involve the parasellar space including Meckel's cave, the lateral aspect of the *sella turcica*, the anterior clinoid process, the optic canal and superior orbital fissure (Graillon et al., 2020), wherefore the management of CSMs (whether surgical or non-surgical) is burdened with CNs, vascular, and endocrinological complications that may render the cure worse than the disease itself. Consequently, CSMs are amongst the most difficult meningiomas to treat. Meanwhile, high precision radiosurgical techniques have demonstrated an excellent safety and efficacy in CSM management, as long term follow-up after SRS have shown high rates of tumour control competing with those obtained after complete resection of the tumour, the surrounding dura and bone, which hardly achieved microsurgically in this complex region (Pollock and Stafford, 2005; Pollock et al., 2013).

In this perspective, careful patient selection for surgical resection is of paramount importance, as is the upfront surgical planning and goal of the surgery. The EOR of CSMs depends on multiple factors, such as tumour extension, ICA involvement, involvement of CNs, tumour consistency and the surgeon's experience. The complete resection including the intra-cavernous portion of the CSM is unsafe. Conversely, a partial resection and decompression of cranial nerves are more likely to be achieved and have become an important goal in CSM surgery: it can be achieved with opening of the roof of the cavernous sinus, peeling of the middle fossa and decompression of the superior orbital fissure and foramina rotundum and ovale, additional to the tumour removal itself.

Altogether, the assessment of the efficacy of the treatments (at large) is challenging due to the indolent growth of the tumour, variations and fluctuations of symptoms even in absence of treatment (oculomotor nerves), lack of histopathological proof when offering SRS as first line treatment, heterogeneity of classifications in the series published, and lack of randomized controlled trials. Here, members of the EANS skull base section and invited renowned experts in the field provide a consensus statement on the overall management of CSMs, including the diagnostic work-up, the different treatment options (whether surgical or non-surgical), as well as adjuvant therapies, summarizing the most recent evidence-based literature on the topic. Eventually, controversies on CSMs management are discussed on a point-by-point basis.

## 2 Methods

A systematic review was conducted according to the Preferred Reporting Items for Systematic Reviews and Meta-Analyses (PRISMA) 2020 guidelines (Page et al., 2021). No registration was required for this study.

On April 12, 2021, we performed a search of literature in Embase, Cochrane Library, PubMed, Google Scholar, and Web of Science. We included literature from January 2000 to December 2020. The following Medical Subject Heading (MeSH) terms were used: “cavernous sinus meningioma” AND/OR “cavernous sinus meningiomas” AND/OR “parasellar meningioma” AND/OR “parasellar meningiomas” AND/OR “epidemiology” AND/OR “radiology” AND/OR “ophthalmology” AND/OR “surgery” AND/OR “endoscopy” AND/OR “microscopy” AND/OR “resection” AND/OR “stereotactic” AND/OR “radiation therapy” AND/OR “radiosurgery” AND/OR “recurrence” AND/OR “survival” AND/OR “outcome”, resulting in a list of 400 articles.

The inclusion criteria were: 1) peer-reviewed research articles, retrospective or prospective in adult patients diagnosed with CSM; 2) histologically confirmed meningioma; 3) number of cases >5 patients; 4) studies written in English, French, German, or Italian language.

**Fig. 1:**
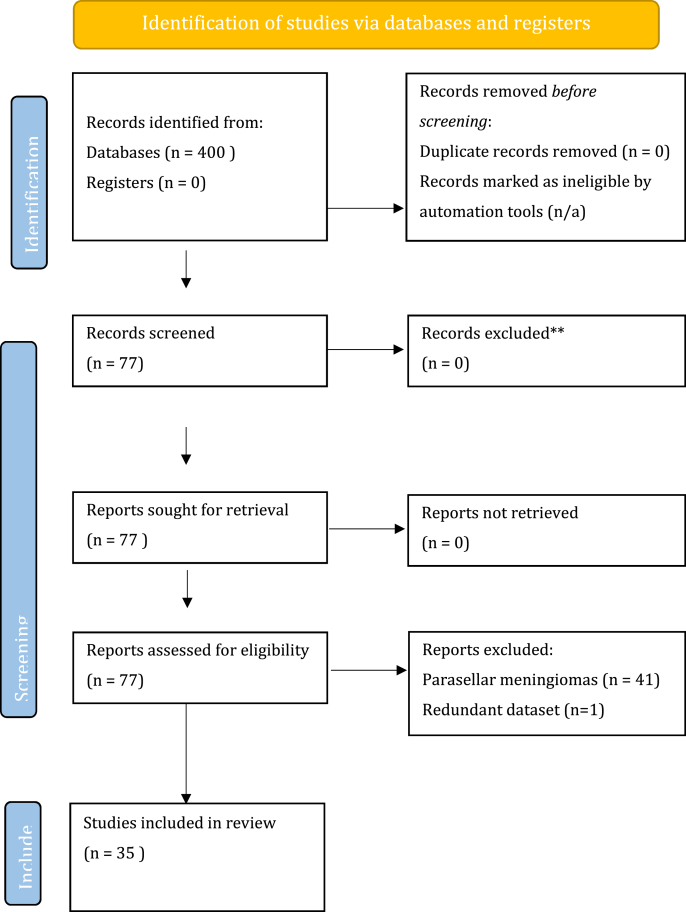
Preferred Reporting Items for Systematic Reviews and Meta-Analyses (PRISMA) flow diagram of the literature review.

Exclusion criteria were: tumours other than CSMs, publications other than original reports and redundant data of a single dataset. Editorials, technical notes, letters, review articles, and case reports were excluded. The titles and abstracts of all the articles were screened independently by MVC and TRM and all the relevant full-text copies were acquired (Fig. 1).

The following data items were considered: 1) study characteristics (author, year, sample size); 2) intervention (surgery, SRS, SRT, other); 3) New/aggravated post-interventional CN deficit; 4) follow-up duration; 5) Mortality; 6) 5-years PFS; 7) 10-years PFS (Table 1).

**Table 1:** Publications retrieved from the systematic review of the literature, resulting in 36 original articles.

*N*	Authors	Year	*N* (Pat)	Intervention	New/aggravated CN deficit (%)	FU duration (median-months)	Mortality (%)	5-years PFS (%)	10-years PFS (%)
1	Roche et al. (Roche et al., 2000)	2000	92	GKS	10	30.5	–	93	–
2	Shin et al. (Shin et al., 2001)	2001	40	SRS	–	42	–	–	–
3	Dufour et al. (Dufour et al., 2001)	2001	31	Surgery ​± ​SRT	–	73,2	–	–	–
5	Lee et al. (Lee et al., 2002)	2002	176	GKS	9	35	–	–	–
6	Nicolato et al. (Nicolato et al., 2002a)	2002	156	GKS	1	48,9	–	87	73
7	Spiegelmann et al. (Spiegelmann et al., 2002)	2002	100	LINAC	0	67	–	–	–
8	Maruyama et al. (Maruyama et al., 2004)	2004	40	SRS ​± ​Surgery	13	47	–	_	–
9	Selch et al. (Selch et al., 2004)	2004	45	SRT	–	36	–	–	–
10	Metellus et al. (Metellus et al., 2005)	2005	36	FRT, GKS	0	63,6	–	100	98
11	Pamir et al. (Pamir et al., 2005)	2005	48	GKS, Surgery	–	39.6–117.6	–	–	–
12	Pollock et al. (Pollock and Stafford, 2005)	2005	49	SRS	–	58	–	94	91
13	Liu et al. (Liu et al., 2005)	2005	174	GKS ​± ​Surgery	–	–	–	98,1	94,1
14	Brell et al. (Brell et al., 2006)	2006	30	FSRT	6,6	50	–	92,5	82,5
15	Sindou et al. (Sindou et al., 2007)	2007	100	Surgery	II: 19; III: 29; IV: 15; V: 24; VI: 17	99.6	5	–	–
16	Hasegawa et al. (Hasegawa et al., 2007)	2007	115	GKS	5	62	0	99	93
17	Jacob et al. (Jacob et al., 2008)	2008	30	Surgery	50	24	–	92	85
18	Pichierri et al. (Pichierri et al., 2009)	2009	147	Open vs close sinus surgery	0	116,4	–	–	–
19	Akutsu et al. (Akutsu et al., 2009)	2009	21	Transsphenoidal surgery	3,50	88,5	0	98,8	92,3
20	Kimball et al. (Kimball et al., 2009)	2009	55	LINAC	0	50	0	95	–
21	Litré et al. (Litre et al., 2009)	2009	100	FSRS	2	33	0	98	93
22	Skeie et al. (Skeie et al., 2010)	2010	100;	GKS	3	82	0	–	–
23	Metellus et al. (Metellus et al., 2010)	2010	53	FRT	–	82,8	0	90,1	75,8
24	Dos Santos et al. (dos Santos et al., 2011)	2011	88	SRS	–	86,8	–	–	–
25	Slater et al. (Slater et al., 2012)	2012	72	FPPRT	15	–	–	92.7	81.2
26	Pollock et al. (Pollock et al., 2013)	2013	115	SRS ​± ​Surgery	25	89	0	–	–
27	Kano et al. (Kano et al., 2013)	2013	272	Surgery ​± ​SRS	10,6	60	0	–	–
28	Zeiler et al. (Zeiler et al., 2012)	2013	30	GKS	3	36,1	0	93	–
29	Correa et al. (Correa et al., 2014)	2014	89	SRS, SRT	3	73	0	–	–
30	Hafez et al. (Hafez et al., 2015)	2015	62	GKS	8	36	0	87	73
31	Haghighi et al. (Haghighi et al., 2015)	2015	57	SRT	28	77	0	–	–
32	Nanda et al. (Nanda et al., 2016)	2016	65	Surgery ​± ​SRS	0	60,8	2	–	–
33	Azar et al. (Azar et al., 2017)	2017	166	GKS, Surgery	3.2	32,4	1	–	–
34	Morisako et al. (Morisako et al., 2018)	2018	9	SX	11	36	0	100	98
35	Hung et al. (Hung et al., 2019)	2019	95	GKS	–	59	–	–	–
36	Gozal et al. (Gozal et al., 2020)	2020	50	Surgery ​± ​RT	24	51.6	0	87.8	–

A PICO question (P: Patient/Problem, I: Intervention, C: Comparison, O: Outcome) was formulated to lead the selection process: the population was defined as adult patients with CSMs, the intervention was any type of procedure performed (stereotactic radiotherapy (SRT) or radiosurgery (SRS), surgery) and outcomes included oculomotor, visual, endocrinological, clinical outcomes, extent of resection, PFS, OS, early and long-term morbidity and quality of life.

The methodological quality of selected articles was evaluated using the Grading of Recommendations Assessment, Development and Evaluation (GRADE) system (Atkins et al., 2004) without masking the authorship of the article.

A task force composed of members of the EANS skull base section along with international experts was created to articulate consensus statements, relying on evidence-based recommendations. The consensus was elaborated after the review of literature and direct discussion among the experts. If randomized blinded trials or prospective matched pair cohort studies were identified, the recommendations were labelled Level A or B, while if only controlled trials or uncontrolled studies were found, the recommendation were labelled Level C or “expert opinion”, respectively (Table 2) (Schunemann et al., 2008).

**Table 2:** Levels of evidence on which recommendations are based. LoE: Level of evidence.

LoE	Definition
A	Sufficient evidence from multiple randomized trials
B	Limited evidence from single randomized trial or other nonrandomized studies
C	Based on expert opinion, case studies or standard of care

**Table 3:** Mandatory (∗) and useful (★) imaging modalities taking place in the assessment of CSM. TOF: time-of-flight; DSA: digital subtraction angiography; CN: cranial nerve; ICA: internal carotid artery; ON: optic nerve.

Radiological Sequences	Assessment	Observation
3D T1 post-gadolinium ∗	Volume of the tumor Dural attachment	Homogeneous & bright enhancement Invasion of surrounding structures
3D T2 anatomical ★	Relations to CNs, ICA and pituitary complex Surgical planification an neuronavigation	Presence of an arachnoid plane Consistency of the tumor
TOF ★	ICA	Narrowing, irregularities and pseudo-aneurysm
FAT Sat★	Course of the ON	Distortion, compression or involvement of the ON
CT scan∗	Bone status	Calcification Hyperostosis Bone erosion
Perfusion CT scan★	Vascular functional reserve	Vascular insufficiency Low flow Ischemia
DSA★	ICA	Narrowing, irregularities and pseudo-aneurysm Cross: compression: vascular reserve

Whenever unanimous responses were obtained, the sentence: “we recommend” was used. In case of divergent opinions, a discussion was undertaken to reach a consensus, using the sentence: “we suggest”. Following a recommendation/suggestion, the literature supporting the assumption is presented, ensued by remarks if necessary.

Finally, the Appraisal of Guidelines for Research & Evaluation (AGREE) Reporting Checklist was performed, to reach the highest possible quality the manuscript (Brouwers et al., 2016).

### 2.1 Assessment of patients with CSMs

#### 2.1.1 Medical history, clinical examination and endocrinological assessment

Patients newly diagnosed with a CSM are commonly symptomatic, as ipsilateral loss of vision is observed in 24%–80% of the cases (DeMonte et al., 1994; George et al., 2003; Jacob et al., 2008; Maruyama et al., 2004; O'Sullivan et al., 1997). Most patients present with at least one progressive neuropathy, including impaired vision, proptosis or disturbed conjugated gaze (DeMonte et al., 1994; Fatima et al., 2020; Gozal et al., 2020; Metellus et al., 2005; Sindou and Alvernia, 2006). This is due to the compression by the tumour, the impingement of the optic nerve (ON) at the level of the falciform ligament and, to a lesser extent, to vascular compromise.

Because the ONs run supero-lateral to the parasellar space*,* their functional assessment is mandatory as soon as CSM tumour is diagnosed and regardless of the treatment decided. Likewise, a comprehensive examination of the extra-ocular muscles, ocular motility and facial sensory changes must be performed, since patients with CSMs frequently present isolated or multiple impairments to the CNs II-VI (Akutsu et al., 2009; Azar et al., 2017; Correa et al., 2014; dos Santos et al., 2011; Fatima et al., 2020; Gozal et al., 2020; Hafez et al., 2015; Haghighi et al., 2015; Hung et al., 2019; Kano et al., 2013; Kimball et al., 2009; Litre et al., 2009; Metellus et al., 2010; Morisako et al., 2018; Nanda et al., 2016; Pichierri et al., 2009; Pollock et al., 2013; Skeie et al., 2010; Slater et al., 2012; Zeiler et al., 2012). Hence, a neuro-ophthalmologist should be systematically included in the early assessment of CSMs; the preoperative work-up must include visual acuity, campimetry, optical coherence tomography, complete examination of extra-ocular motility, corneal reflex, assessment of direct and consensual pupillary reflexes as a part of the baseline examination (Blanch et al., 2018; Danesh-Meyer et al., 2015; Garcia et al., 2014; Jacob et al., 2009; Tieger et al., 2017) (Table 4). The presence of trigeminal neuropathic pain and/or trigeminal neuralgia, secondary to compression of V_1_, V_2_ and/or V_3_ should also be assessed and treated accordingly.

**Table 4:** Non-radiological Baseline assessment of newly discovered cavernous sinus meningioma. TSH: thyroid-stimulating hormone.

Category	Pre-operative assessment
Ophthalmology/Neurology	Direct/indirect pupillary reflexes optical coherence tomography, complete examination of extra-ocular ocular motility
Endocrinology	Prolactin, gonadotropins, insulin-like growth factor 1, TSH and free T4, as well as 8 a.m. cortisol and 24-h urine-free cortisol

There are no clear-cut recommendations regarding preoperative assessment of endocrinological function in patients with CSMs. However, assessment of the pituitary function is essential, particularly in the case of CSMs invading the CS medially, or when there is contact between the tumour and the pituitary complex (DeMonte et al., 1997; Giammattei et al., 2020) or when dislocation of the pituitary stalk is seen on pre-operative imaging. Should it be the case, we recommend measurements of prolactin, gonadotropins, insulin-like growth factor 1 IGF-1, thyroid-stimulating hormone and free T4 as well as 8 a.m. cortisol and 24-h urine-free cortisol.

Hyperprolactinemia is the most frequently encountered endocrinological disturbance, whereas hypopituitarism remains rare and cases of diabetes insipidus or syndrome of inappropriate secretion of anti-diuretic hormone are very rare (Bassiouni et al., 2006; Ciric and Rosenblatt, 2001; Fujio et al., 2017; Jallo and Benjamin, 2002; Komotar et al., 2012; DeMonte et al., 1997) (Table 5).

*1. The EANS task force recommends that patients with newly diagnosed CSM undergo a complete history and clinical examination by a neuro-ophthalmologist, including visual acuity and fields, oculomotricity, corneal reflex and facial sensory changes. Furthermore, a thorough endocrinological assessment with complementary blood tests should be performed to rule out any preoperative endocrinological deficit whenever the pituitary complex is involved (****Level C****).*

**Table 5:** The Levine-Sekhar grading system includes history of previous radiotherapy/radiosurgery, the degree of vessel encasement seen on pre-operative magnetic resonance imaging and the presence cranial nerve palsy on clinical examination. The final scores corresponds to a grade of resection. RT: Radiotherapy; RS: Radiosurgery; CN: Cranial nerve.

Category	Variable	Presence	Absence	Possible score	Resection score	Corresponding grade	EOR (% totally resected
History	Previous RT/RS	1	0	0–1	0	0	**90%**
Imaging studies	Vessel encasement Multiple fossa involvement	1 1	0 0	0–2	1–2	I	**60%**
Physical examination	CN palsy III V VI	1 1 1	0 0 0	0–3	3–4	II	**40%**
Total				0–6	5–6	III	**13%**

#### 2.1.2 Radiological assessment

The basic imaging work-up includes a cerebral magnetic resonance imaging (MRI) with angiographic sequences, as well as a brain computed tomography (CT) scan. In particular, 3D T1 post-gadolinium sequences, 3D T2 anatomical sequences (CISS sequence for further assessment of the trajectory of the CN in the lateral wall of the CS and cisternal segments) to locate the position of the lateral wall of the CS inside the tumour and time-of-flight (TOF) angiographic sequences must be obtained to assess the tumour and its relations to the adjacent neurovascular structures (Heth and Al-Mefty, 2003; George et al., 2003). Fat-saturated (fat-sat) sequences are required to assess the course of the ONs with precision. A CT scan should be performed to evaluate the presence of calcifications within the tumour as well as associated hyperostosis (need for thin slides bone window CT of the skull base). Alternatively, CT scan can be performed only when surgery is indicated, to avoid unnecessary radiation. Perfusion CT scan can be considered if there is doubt regarding the patency of the cavernous ICA (Alzhrani et al., 2019; Corniola et al., 2021).

Alternatively, some authors perform preoperative digital subtraction angiography (DSA) with balloon occlusion test to evaluate the ICA patency as well as tolerance for ICA occlusion, since strokes due to scheduled or accidental peri-operative ICA closure are reported in up to 5% of the cases (Cusimano et al., 1995; De Jesus et al., 1996; Heth and Al-Mefty, 2003) (Table 3). Assessment of the patency or stenosis of the cavernous segment of the ICA is paramount in cases of CSMs: tumours presenting with a 360° involvement of the cavernous ICA and with stenosis of the cavernous ICA have been associated with increased risk of carotid injury if complete microsurgical resection is attempted.

**Fig. 2:**
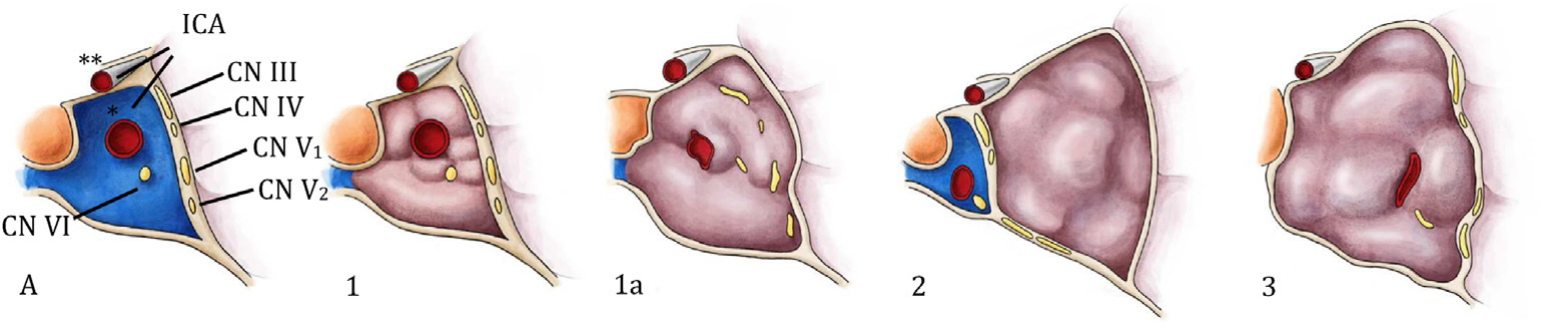
The anatomy of the cavernous sinus, coronal view(A) and the three growth patterns CSMs. 1): The tumour is confined to the cavernous sinus which is not distorted and venous blood flow is partially maintained. This situation is unfavourable to surgery. 1a) The tumour grows into the cavernous sinus, encircling the cranial nerves passing by and strangulating the cavernous segment of the internal carotid artery. The cavernous sinus is distorted and occluded. This situation is unfavourable to surgery. 2): The tumour grows laterally to the cavernous sinus, leaving the neurovascular structure free medially. This scenario is favourable to open surgery. 3): The tumour grows medially to the cranial nerves, pushing them laterally, into the lateral capsule. The cavernous sinus is distorted and enlarged laterally. This situation is favourable to transnasal endoscopic surgery. CN: cranial nerve; ICA: internal carotid artery, ∗ cavernous segment; ∗∗ supra-clinoid segment. Drawing author: Lisa Cuthbertson.

The configuration of the pathological anatomy of the CS is essential in the treatment decision. The pre-operative imaging often demonstrates one of three main growth patterns, and three scenarios can be seen (Fig. 2) (Dietemann et al., 1998; Kehrli et al., 1998):

1) The lesion arises from the arachnoid granulations inside the CS and grows inwards, inside the CS, encasing its neurovascular contents (*unfavourable to surgery*);
2) the CSM arises from the lateral wall of the CS and grow toward the temporal lobe., pushing the neurovascular structures medially to the lesion (exophytic CSM, *favourable for craniotomy*) and;
3) the lesion arises from the medial wall of the CS, grow towards the sinus, pushing everything laterally and splaying the CNs, thereby opening the space inbetween them (*favourable for transnasal endoscopic - controversial*) or encasing everything inside (*unfavourable to surgery*). This is often a spheno-cavernous meningioma infiltrating the sinus and extending into the sphenoid sinus.

The anatomical relationship between the tumour and the superior orbital fissure is also important, since CSMs involving the posterior aspect of the CS might be surgically resectable, whereas the lesions infiltrating the superior orbital fissure, where the CNs II-VI converge and lay on top of each other, may be too risky for any surgical therapy. In the latter case, non-surgical therapy should be envisioned. Yet, only partial CN palsies of III-VI are a negative predictive factor in the surgical outcome, while complete CN palsy does not speak against surgery (is a good indication for surgery).

*2. The EANS task force recommends that all patients with a newly discovered lesion compatible with a CSM undergo cerebral MRI with 3D T1 post-gadolinium sequences, 3D T2 anatomical sequences, time-of-flight (TOF) angiographic sequences and Fat sat sequences to assess the lateral/upward/posterior extension of the tumour in the parasellar area, the involvement of CNs II-VI, the overall anatomy of the region and the vasculature, in particular the cavernous segment of the ICA. A cerebral CT scan should also be performed to assess the presence of hyperostosis in the parasellar area when surgery is indicated. The hyperostosis can be seen with sufficient accuracy in T2-weighted images, whenever a CT scan cannot be performed (whatever the reason). As part of the preoperative planning, digital subtraction angiography (DSA) with balloon occlusion test to evaluate the ICA patency as well as tolerance for ICA occlusion can be undertaken (****Level C****).*

### 2.2 General management of CSM

**Fig. 3:**
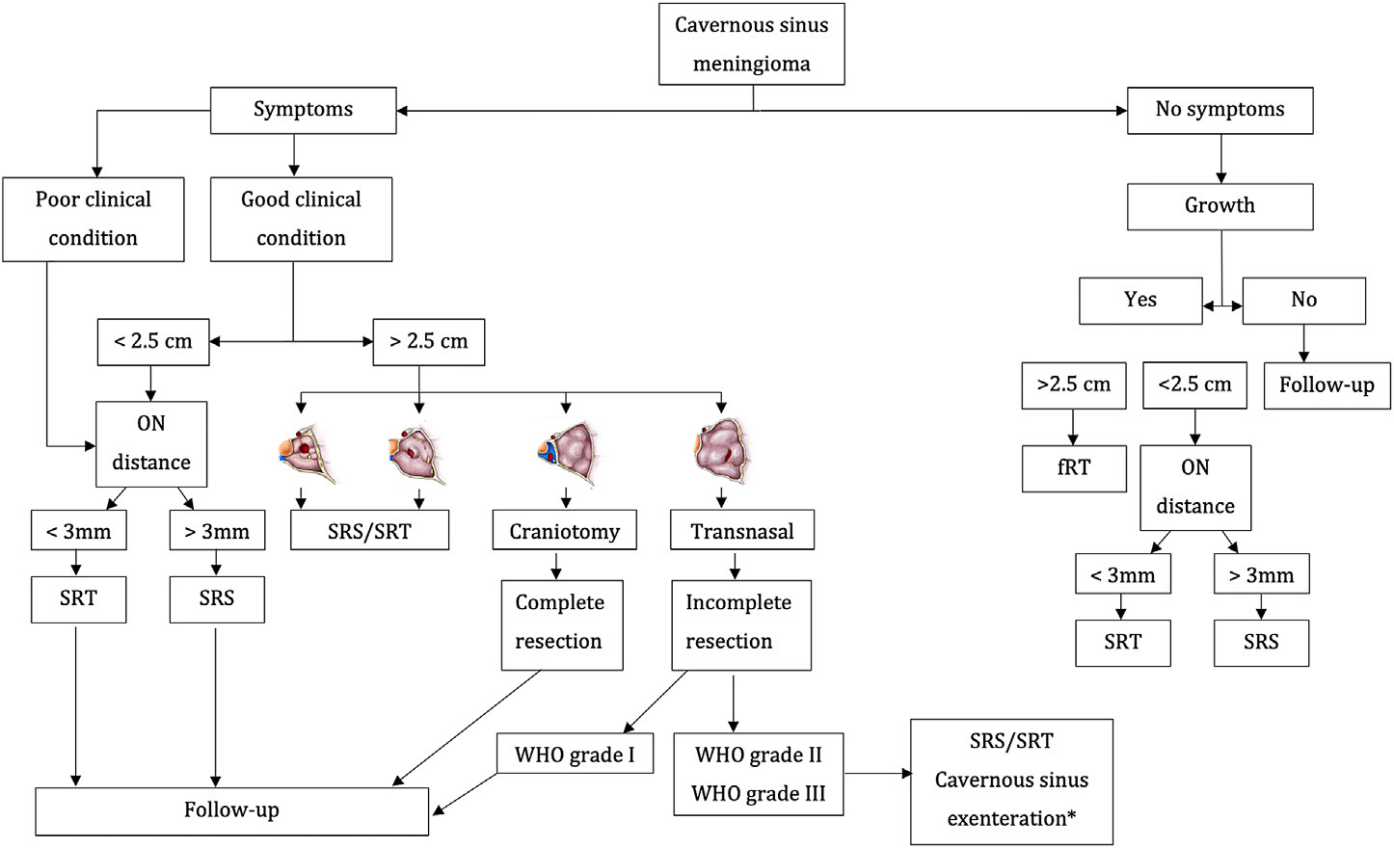
Proposed management of cavernous sinus meningiomas, according to the presence of symptoms, extent of tumour and extent of resection. The different management strategy are mainly based on the presence of symptoms, patient general condition and presence of growth on serial imaging. ON: Optic nerve; SRS: Stereotaxic radiosurgery; SRT: Stereotaxic radiotherapy; fRT: Fractionated radiotherapy; WHO: World Health Organization.

CSMs can be managed conservatively, with surgery, radiosurgery or using radiotherapy. Their overall management is summarized in simplified and standardized guidelines published elsewhere by the European Association of Neuro-Oncology (EANO) (Goldbrunner et al., 2016). Here, we provide a consensus on the management strategy for CSMs: their critical location and slow evolution along with their frequent presentation with CN deficits (mostly involving visual of oculomotor functions) despite their benign nature, requires specific adaptations of the general management of intracranial meningiomas, summarized in Fig. 3.

The therapeutic attitudes regarding CSMs can range from only conservative to an aggressive surgical tumour removal with CS exenteration and EC-IC bypass surgery. The treatment decision should consider:

1) the clinical symptoms and signs on presentation;
2) the size of the tumour and its consistency;
3) the biological behaviour of the meningioma;
4) the preoperative work up: MRI, DSA, balloon test occlusion;
5) the experience of the multidisciplinary team in charge of the patient.

In any case, the decision to offer one treatment over another should be taken by a multidisciplinary board, where experienced surgeons and physicians meet and share their expertise in view of the best possible outcome based on a case-by-case discussion.

#### 2.2.1 Patient counselling

Any treatment for CSM is never to be taken lightly, even when non-surgical therapy is chosen. Therefore, patient counselling is paramount. As skullbase meningiomas usually evolve slowly, one should never rush into a treatment without taking as much time as necessary to inform the patient (Giammattei et al., 2021). The neurosurgeon and radiotherapist must ascertain that the patient and close relatives perfectly understand the stakes, the risks and benefits of the therapy, pertaining to CN dysfunction and vascular injury. The surgical strategy and the non-surgical alternatives should be discussed thoroughly. The treating physician should explain the possible complications, like the occurrence of oculomotor disturbances and trigeminal dysfunction, including hypoesthesia and/or facial pain, as well as their impact on quality of life (QoL). The risk of vascular injury requiring surgical repair or endovascular occlusion should also be clearly mentioned. Finally, the mortality rate of the surgical treatment must be discussed.

Regardless of the presence of CN impairment, the QoL can be reduced following meningioma surgery, with a certain proportion of patients not returning to work or presenting with mood disorders (Corniola and Meling, 2021). This should be stated to the patient and discussed.

*3. The EANS task force recommends patient counselling prior to the treatment of a CSM in order to extensively discuss the risk and benefits of any surgical or non-surgical treatments and natural history of the disease, especially if asymptomatic. Perspectives in terms of QoL, functional impairment and mortality should also be openly discussed (****Level C****).*

#### 2.2.2 Decision-making process

Treatment of CSMs ranges from purely conservative to aggressive surgical removal. Regarding conservative therapy, the average annual growth rate is between 0.7 and 3 mm; large size and younger age are identified factors favouring growth, whereas calcifications seem to protect against tumour progression (Olivero et al., 1995; Go et al., 1998; Kuratsu et al., 2000; Niiro et al., 2000; Nakamura et al., 2003; Yoneoka et al., 2000; Herscovici et al., 2004; Yano et al., 2006).

The GTR rate offered by microsurgery varies from one series to the other, ranging from 17% to 82%, while up to 70% of patients undergoing CSM surgery experience post-operative new CN deficit. The overall surgical mortality varies from 1 to 16% (De Jesus et al., 1996; O'Sullivan et al., 1997; Cusimano et al., 1995).

Radiosurgery provides a 95% overall tumour control rate over more than 7 years of follow-up, with complications rate/worsened neurological outcome ranging from 3 to 6% (Metellus et al., 2005). About 60% of the patients show an improved clinical outcome, either with gamma knife radiosurgery (GKRS) or with conventional RT and 30% are clinically unchanged after therapy, with an overall morbidity of 1.6%–11.5% (Dufour et al., 2001; Metellus et al., 2005; Maire et al., 1995; Vendrely et al., 1999; Maguire et al., 1999; Nutting et al., 1999). From a purely physical point of view, conventional RT is exposing much more normal tissue to a significant amount of radiation, carrying a risk of carcinogenesis (2%), radionecrosis and cognitive decline. Regarding SRS in particular, the tumour control rate is up to 84%, the re-treatment rate is 12% and the complication rate 6% (CN neuropathies, symptomatic/non-symptomatic ICA occlusion) (Skeie et al., 2010).

To help with the decision, Levine et al. (1999) developed a pre-operative scale including six variables known as negative predictive factors in the surgical outcome: 1) previous radiotherapy/radiosurgery; 2) vessel encasement; 3) multiple locations; 4) CN palsies of III-V-VI. Using a simple binary scale, the authors can predict the EOR, ranging from 90% to 13%, corresponding to a score of 0 (minimum) or 6 (maximum) **(**Table 5**)**.

Another score to predict the surgical outcome in the perspective of extra-ocular motility has been brought by Hirsch et al. (1993), based on the pre-operative radiological assessment of vessel encasement on MRI (Table 6). Contrary to the Levine-Sekhar score, which considers only the surgical perspective in terms of EOR, Hirsch et al. (1993) focus on the functional outcome, which is more in line with the current philosophy of functional preservation. Both scales are based on the analysis of preoperative cerebral MRI, in particular the 3D T2 and TOF sequences. However, these two classifications should be considered cautiously since they have been published more than 20 years ago, were based on small series, and in a period where aggressive surgery was central to the CSMs treatment. Still, they can be considered as a helpful base to balance the treatment decision even in the modern era.

**Table 6:** The Hirsch grading system relies on the pre-operative radiological assessment of the encasement of the cavernous segment of the internal carotid artery, predicting the surgical resectability and the outcome, in terms of post-operative extraocular motility. cICA: Cavernous segment of the internal carotid artery; EOM: Extraocular motility.

Category	Definition	Post-operative recovery of EOM
I	cICA not completely encircled. Easy to dissect from the vessel without injury, sacrifice or grafting.	84%
II	The cICA is completely encircled, w/o stenosis. Successful dissection of the tumour w/o injury in 61%	36%
III	The cICA is encircled and narrowed and the dissection carries high risk of vascular injury.	

Aside from cavernous ICA encasement and the presence of other negative surgical predictors, tumour consistency is of great importance, as soft tumours are better candidates for surgery than firm ones. However, meningioma consistence is very difficult to predict preoperatively (Sauvigny et al., 2020; Yao et al., 2018).

### 2.3 Conservative management of CSM

In the case of incidental CSMs, tumour progression dynamics should be evaluated by serial contrast-enhanced MRIs, initially at six months and then once yearly if the patient remains asymptomatic. Studies on the natural evolution of CSMs report that a certain proportion of incidentally discovered CSMs will eventually grow during follow-up and become symptomatic. It is also known that CSMs presenting with initial neurological impairment often have symptomatic progression over time (Bindal et al., 2003; Nakamura et al., 2003). After 5 years of annual follow-up of a non-growing, asymptomatic CSM, the interval can be doubled. In the specific case of elderly patients with a limited life expectancy (1–2 years), monitoring may be omitted (Goldbrunner et al., 2016).

Some progestin-induced CSMs (cyproterone acetate, chlormadinone acetate and nomegestrol acetate) reduce in size after treatment cessation. In those cases, treatment should be avoided (Bernat et al., 2015; Voormolen et al., 2021).

*4. The EANS task force recommends that conservative treatment with serial imaging follow-up should be proposed in patients with a newly diagnosed asymptomatic CSM that has no mass effect on the adjacent temporal lobe (****Level C****). Whenever the CSM is suspected to be progestin-induced, hormonal treatment should be discontinued at first.*

### 2.4 Non-conservative treatment of CSM

Recent mid-to-long-term follow-up data on the natural history of CSMs managed conservatively show an average tumour growth rate of 1.34 cm^3^/year and a mean tumour doubling time of 13.6 years (Nakamura et al., 2003). Therefore, it seems reasonable to advocate at least some form of active treatment of CSMs, ranging from primary SRS/SRT to surgical resection aiming to relieve mass effect and leaving remnant to adjuvant therapy whenever necessary. This latter option ensures a speedy relief from the mass effect without carrying the morbidity/mortality of a maximalist resection.

#### 2.4.1 Radiation therapy and stereotactic radiosurgery

Here, we summarize the non-surgical therapies in a single chapter for the sake of clarity, but the reader must keep in mind that RT and SRS may not be equivalent. However, it should be noted that grouping RT and SRS together is questionable, mostly because the radiobiology of these two therapies is very different (models for equivalent doses are just models).

SRS, SRT and RT (either single-dose or fractionated) are indicated in the case of small tumours or whenever surgery is not feasible, especially in the case of asymptomatic or pauci-symptomatic CSMs (Lee et al., 2018; Stafford et al., 2001; Starke et al., 2012). Radiation therapy, be it by SRS, SRT, or f-SRT, has similar rates of tumour control and improvement of pre-existing CN deficits as open surgery (Tishler et al., 1993). This affirmation, based on the results of a series of 62 patients, was published in 1993 and should therefore be interpreted with caution in the modern era, since the conclusions drawn in the ‘90s are mostly based on non-inferiority studies rather than randomized controlled trials.

The tumour control rate after SRS/RT using a median margin dose to the lesion of 13–15 Gy is up to 95% (Akyoldas et al., 2020; Cohen-Inbar et al., 2018; Hasegawa et al., 2007; Kaspera et al., 2015; Lee et al., 2002; Montaser et al., 2017; Nicolato et al., 2002b; Park et al., 2018; Santacroce et al., 2012; Sheehan et al., 2014; Skeie et al., 2010; Spiegelmann et al., 2010; Starke et al., 2014; Williams et al., 2011), with a complication rate between 3 and 12% (Dufour et al., 2001; Maguire et al., 1999; Maire et al., 1995; Metellus et al., 2005, 2010; Nutting et al., 1999; Skeie et al., 2010; Vendrely et al., 1999). In case of fractionation, the total dose is higher, but the peak dose per fraction is dramatically reduced: that is the reason why fractionation is required when the tumour is at the proximity of the ON. Radiation-induced optic neuropathy is reported in up to 3% patients, while other radiation neuropathies are reported in <2% of patients, when it comes to the parasellar area. SRS seems to be safer than open surgery in the case of small, circumscribed lesions.

The radiation dose administered to the tumour must be limited whenever the meningioma abuts against, encases, or compresses the optic pathways. In SRS, the safe distance to the ONs or chiasm varies from 2 to 4 mm (Duma et al., 1993; Nicolato et al., 2002a; Maruyama et al., 2004; Pollock et al., 2013; Shrieve et al., 2004; Stafford et al., 2003), while most studies reporting on RT manage tumours in contact with the optic pathways (Leroy et al., 2018). A study reporting multi-session radiosurgery (2–5 daily fractions) for large meningiomas close to the optic apparatus (21–25 Gy/3–5 fractions) shows a local control of 93–95% at 5 years, with reduced visual toxicity (Marchetti et al., 2016).

RT for brain tumours is known to cause hormone deficiency in some patients, including growth hormone (GH), thyroid hormones, adrenocorticotropic hormone and gonadotropins. The frequency, rapidity of onset and the severity of these abnormalities correlate with the total radiation dose delivered, as well as the fraction size, younger age at irradiation, prior pituitary compromise by tumour and/or surgery and the length of follow-up (Darzy and Shalet, 2005). The GH axis is the most vulnerable to radiation damage, and isolated GH deficiency can occur after doses as low as 18 Gy. Furthermore, the frequency of GH deficiency can reach 50% within 3–5 years of cranial irradiation with doses of 30 Gy (Darzy, 2009). GH deficiency is associated with an increased cardiovascular risk and a physiologic substitution seems to have beneficial effects on body fat mass, cholesterol profile and blood pressure (Meling and Nylen, 1996). TSH and adrenocorticotropic hormone deficiency occur in 3–6% of patients after conventional irradiation (30–50 Gy)(Darzy, 2009). Regular testing is therefore mandatory to ensure timely diagnosis and early hormone replacement therapy.

In the case of CSM enclosed within the parasellar lodge, SRS is advocated. If there is uncertainty regarding the histology, a percutaneous biopsy through the foramen ovale can be discussed. However, it could be negative if the lesion is located anteriorly and/or shows hard consistency (Sindou et al., 1997, 2012; Messerer et al., 2012; Arishima and Sindou, 2010).

#### 2.4.2 Predicting failure of non-surgical therapy

Regarding SRS, a tumour volume exceeding 15 cm^3^ or 3 cm of diameter (Lee et al., 2002), non-WHO grade I histology and male sex (Kuhn et al., 2013) are independent predictors of treatment failure. In the same way, an unexpected high tumour shrinkage after SRS should alert the clinician about a non-meningiomatous origin of the disease and potential aggressiveness of the lesion (e.g. hemangiopericytoma). In this perspective, tumour control aiming at volume stabilization should be the rule, rather than volume reduction. Pertaining to RT, the cut-off diameter seems to be near 5 cm (Connell et al., 1999; Maire et al., 1995). For both SRS and RT*,* planning failure is also to be considered (Tripathi et al., 2020). Lastly, rapidly symptomatic lesions or lesions with unusual imaging features should be considered for biopsy and/or decompression.

5. The EANS task force recommends that SRS or SRT (either single-dose or fractionated) should be considered in the following cases, insofar as the distance to the ON is superior to 3 mm (**Level C**):

- *Asymptomatic, > 40 years old patients with a purely intracavernous CSMs <2.5 cm showing growth on serial imaging after initial conservative treatment;*

- *Asymptomatic patients with partly extracavernous CSMs showing growth on serial imaging after initial conservative treatment;*

- *Symptomatic patients with CSMs <2.5 cm, provided that the symptoms are not related to ON compression*

- *Symptomatic patients with partly extracavernous CSMs in whom surgery is contraindicated.*

*The EANS task force recommends that fractionated RT should be considered in cases that warrant treatment (see above) if the distance to the ON is less than 3 mm and the ipsilateral visual function is good (****Level C****).*

#### 2.4.3 Surgery

During the early years of skull base surgery, aggressive tumour removal was advocated, but the results were often discouraging pertaining to rates of GTR and morbidity (Gozal et al., 2020; Raheja and Couldwell, 2020). Today, a more conservative approach to CSMs is favoured (cytoreductive surgery), focusing more on preservation of function and QoL (Goldsmith et al., 1994; O'Sullivan et al., 1997).

Whenever surgery is indicated due to ON, brainstem or temporal lobe compression, CSMs can be approached surgically either trans-cranially or trans-nasally. Yet, the two options are not equal. The indications for EEA of cavernous meningiomas remain limited compared to transcranial approaches: whenever surgery is indicated due to ON, brainstem or temporal lobe compression, CSMs should be approached trans-cranially. In case of ON compression or oculomotor dysfunction, EEA have been used for CN decompression.

During surgery, direct stimulation of the lateral wall of the tumour/CS using neurostimulation should be performed in all cases (Hariharan et al., 2018; Kaspera et al., 2015; Kawaguchi et al., 1995; Sekhar and Moller, 1986; Son et al., 2012). Furthermore, neurophysiologic monitoring should be carried out, using motor and somatosensory evoked potentials. Additionally, CN III, IV and VI may be monitored. Lastly, doppler ultrasound is used for early identification and preservation of the cavernous segment of the ICA.

Standard anaesthesiology techniques can be used during CS surgery. However, the anaesthesiologists should be aware of the risk of venous bleeding and vagal reaction following ON manipulation during surgery. Additionally, anaesthesiologists should be aware of the potential for bradycardia during resection of tumour extending into Meckel's cave due to the trigeminal reflex. The CO_2_ pressure level should be kept under 3.5 ​mmHg to reduce venous congestion. In the same vein, the head should be elevated (up to 30°) to reduce venous congestion.

Besides the general surgical complications, surgery in and around the CS is associated with a risk of transient/permanent CN function impairment. Yet, the ON function may be significantly improved after surgery (Newman, 2007). Often, temporary CN dysfunction is observed after surgery, with significant recovery in the first 3–6 months after surgery. It must be kept in mind that all CN palsies are not equal: a palsy of CN III has a completely different significance/impact than a palsy of CN VI; CN VI palsy can be compensated by ophthalmological surgery, whereas palsy of the CN III is equal blindness and must be avoided at any price.

#### 2.4.4 Surgical approaches to the cavernous sinus

**Table:** 

Regular intra-dural approach via a fronto-temporal craniotomy
Hakuba approach: orbitozygomatic infratemporal combined epi-subdural approach (Hakuba et al., 1982).
Dolenc approach (Dolenc, 1983); extradural resection of the anterior clinoid process, peeling of the dural to offer an “interdural approach”
Kawase approach (anterior transpetrosal transtentorial) (Kawase et al., 1991) (Kawase)
Endoscopy-assisted transcranial approaches to the CS from Radovanovic (Andrade-Barazarte et al., 2019) and endoscopic endonasal approaches (Cappabianca et al., 2008; Truong et al., 2018; Koutourousiou et al., 2017).

Sindou et al. (2007) reported a series of 100 patients with CSMs managed by surgery as stand-alone therapy. In their series, the mortality rate was 5% and the permanent neurological morbidity (other than CN palsy) was 2%. However, with respect to CN dysfunction, 19% of patients had a new or aggravated visual deficit, 29% presented impaired extra-ocular motility, and 24% of patients had a disturbed trigeminal function. Whenever the resection was continued into the CS itself, the complication rate increased significantly (Sindou et al., 2007). Pertaining to the EOR, 12% of patients had GTR, 28% of patients had subtotal resection (STR) including part of the intra-cavernous part of the tumour and 60% had subtotal resection of the extra-cavernous portion of the tumour. Tumour regrowth was reported in 13% of the patients who underwent STR (Sindou et al., 2007). However, these results should be interpreted cautiously, since other studies found much higher recurrence rates when observing patients with prolonged follow-up (Mathiesen et al., 1996). Furthermore, Shaffrey et al. (1999) showed that CSMs encasing the ICA not only narrow the lumen, but tend to infiltrate the vessel wall; in that perspective, attempted radical resection of the lesion is fraught with danger. Altogether, these results indicate that satisfactory tumour control can be achieved with surgery by experienced surgeons, although functionally impairing complications are nevertheless not uncommon (Larson et al., 1995).

CSMs have been approached using different transsphenoidal microsurgical corridors to biopsy the tumour and decompress the bony wall of the cavernous sinus, favouring CN recovery and optimizing the efficacy of radiotherapy (*e.g.* interposition of fat graft between tumour and pituitary to preserve function and allow early radiation (Taussky et al., 2011),).

The development of extended approaches allowed for further possibilities (Alfieri and Jho, 2001), and a variety of transsphenoidal, transmaxillary, transmaxillo-sphenoidal, transethmoidal and transspheno-ethmoidal microsurgical approaches have been suggested to remove lesions involving the anterior portion of the CS, exophytic sellar and supra-sellar components of the CSMs. This expanded our armamentarium to achieve decompression of the ONs and/or chiasm, pituitary gland/stalk, and to obtain additional bone removal over the cavernous sinus and optic canal, ensuring minimal retraction of neurovascular structures (Couldwell et al., 1997; Das et al., 2001; Fahlbusch and Buchfelder, 1988; Fraioli et al., 1995; Hashimoto and Kikuchi, 1990; Inoue et al., 1990; Kitano and Taneda, 2001; Lalwani et al., 1992; Sabit et al., 2000; Honegger et al., 1993; Akutsu et al., 2009; Beer-Furlan et al., 2020; Sivakumar et al., 2019; Graillon et al., 2014).

The narrow corridors offered by EEAs do not allow safe and adequate exposure of the lateral aspect of the CS and are ineffective for tumour portions that extends beyond the limits of the Meckel's cave. Still, GTR is rarely - if ever - achieved, especially when the surgeon is not experienced or whenever the tumour is firm, fibrous and adherent to the adjacent structures.

### 2.5 Extent of resection and its intra-operative assessment

In the specific case of CSMs, the current trend is rather to decompress surgically and to proceed with adjuvant therapy. In this perspective, the Simpson grading score is less relevant (Schwartz and McDermott, 2020). DeMonte et al. (1994) attempted to update the Simpson scale in a series of CSMs by creating a scale from unpublished data of Kobayashi, based on the surgeon's intra-operative subjective evaluation of the EOR (Table 7). Aside from the EOR as prognostication tool, emergent grading systems such as the Copenhagen Protocol, based on microscopic analyses of resection margins and ^68^Ga-DOTATOC PET may result in improved overall prognostication and therefore reveal useful in the specific context of CSMs, where the EOR is extremely difficult to estimate (Haslund-Vinding et al., 2021). Overall, PET imaging in meningiomas is not yet considered clinical routine, but certainly has growing clinical potential as reported and well summarized by the RANO/PET Group a few years ago (Galldiks et al., 2017)

*6. The EANS task force recommends proceeding to surgery in the following cases (****Level C****):*

**Table:** 

Biopsy/Decompression	Maximal safe resection	Aggressive surgery/Cavernous sinus exenteration
*Atypical lesion* *Unclear diagnosis* *Rapidly symptomatic lesions or unusual radiology* *Alternatively,*^*68*^*Ga DOTATATE-PET* (Klingenstein et al., 2015) *or 68Ga DOTATOC-PET* (Haslund-Vinding et al., 2021) *can be performed (high sensitivity)*	*Symptomatic, partly extra-cavernous CSM* *Young patients (< 40 years) with asymptomatic, but growing CSM might be considered for surgery, if patient agrees.* *Progressive visual loss due to ON compression*	*Complete visual loss* *Complete ophthalmoplegia* *Complete visual loss and complete ophthalmoplegia* *Recurrence after radiation* *Aggressive tumour histology/behaviour*

**Table 7:** Assessment of the extent of resection of cavernous sinus meningiomas according to the modified Kobayashi tumour removal grading system as described in DeMonte et al. (1994). SRS: stereotactic radiosurgery; SRT: stereotactic radiotherapy.

Modified system of Kobayashi et al.	
Grade I	Complete microscopic removal of tumour & dural attachment with any abnormal bone
Grade II	Complete microscopic removal of tumour with diathermy coagulation of its dural attachment
Grade IIIA	Complete microscopic removal of intra- & extradural tumour without resection or coagulation of its dural attachment
Grade IIIB	Complete microscopic removal of intradural tumour without resection or coagulation of its dural attachment or any of its extradural extensions
Grade IVA	Intentional subtotal removal to preserve CNs or blood vessels with complete microscopic removal of attachment
Grade IVB	Partial removal leaving tumour ≤10% in volume
Grade V	Partial removal leaving tumour >10% in volume, or decompression with or without biopsy

### 2.6 Adjuvant therapy

In the series of Sindou et al. (2007), extensive craniotomies with orbitozygomatic osteotomies were performed in 97% of the patients, with proximal control of the ICA in 65% of the cases. The para-clinoid segment of the ICA was exposed in 81%; a second-stage surgery was performed in 27% of the patients, to achieve resection of the posterior petroclival extension of the meningioma. However, GTR was only achieved in 12% of the patients. Surprisingly, regrowth during follow-up (mean: 8.3 years) was noted in only 13% of the patients, showing that 1) RT can be reserved to the minority of patients showing post-operative tumour growth and 2) that only a minority of tumours grow during long-term follow-up. Again, these conclusions are based on a single series and should be interpreted with some care. In some cases, panel analysis following tumour biopsy can be performed, to identify potential target therapy, such as it is the case with m-TOR (Everolimus).

*7. The EANS task force recommends considering adjuvant SRS or f-SRT after subtotal surgical resections (****Level C****) whenever growth of residual tumour is observed during follow-up through tumour remnant volume analysis.*

## 3 Follow-up of CSM

The aim of the post-interventional follow-up is to detect any tumour remnant evolution or meningioma recurrence as well as to identify early/late treatment-related complications. The basis for recommendations for post-treatment patient follow-up is weak and most studies use variable follow-up protocols. Consequently, the recommendations published by the EANO are based more on the consensus opinion of experts than on scientific evidence (Goldbrunner et al., 2016).

Although the majority of patients with CSMs are observed or treated with non-surgical procedures, an extended multidisciplinary follow-up is mandatory. As an example, while panhypopituitarism is rare in CSMs, their treatment can be the cause of significant pituitary disturbances. Whenever SRS or f-SRT are given, either as first-line or adjuvant therapy, there is a risk of interference with the normal pituitary function with some patients requiring life-long hormonal replacement (Auernhammer and Vlotides, 2007). Approximately 42% of patients will develop hypopituitarism within 7 years after SRS or RT, and up to 70% of patients within 17 years (Hoybye et al., 2001; Laws et al., 2004; Pollock et al., 2008; Sheehan et al., 2003, 2005a, 2005b). Pollock et al. (2013) reported permanent complications in up to 12% of the patients, including trigeminal dysfunction, diplopia, ischemic stroke due to ICA occlusion and hypopituitarism, with a 2-, 5- and 10-years rates of 7%, 10%, and 15%, respectively, stressing the need for a long-term, comprehensive follow-up. Lastly, Correa et al. (2014) showed that f-SRT and SRS carries similar rates of clinical and radiological improvements.

The follow-up should be performed by an experienced neurosurgeon and integrated in the perspective of a multidisciplinary team involving radiation therapists, oncologists, ophthalmologists and endocrinologists (if necessary). The interval between follow-up visits can vary widely, depending on treatment modality, the EOR (in case of surgical management), the dose (in case of SRS/f-SRT), the initial size of the lesion, the patient's age, and general and the neurological condition.

*8. Patients diagnosed with CSMs should undergo an appropriate follow-up, including oncological, ophthalmological, endocrinological, neurological and neurosurgical assessment, and according to the latest EANO guidelines (****Level C****).*

### 3.1 Strengths and limitations

This manuscript is the result of an international collaborative effort reflecting, on the one hand, a detailed literature review and, on the other hand, the experience accumulated by the authors over the past years. However, the systematic review on a complex pathology such as CSMs is a real challenge, since high level of evidence is undoubtfully very difficult to create. Therefore, we gathered surgeons with various background and countries to provide clear and as much objective as possible guidelines, under the authority of the EANS skullbase section.

## 4 Summary

### 4.1 Medical history, clinical examination and endocrinological assessment

The EANS task force recommends that patients with newly diagnosed CSM undergo a complete history and clinical examination by a neuro-ophthalmologist, including visual acuity and fields, oculomotricity, corneal reflex and facial sensory changes. Furthermore, a thorough endocrinological assessment with complementary blood tests should be performed to rule out any preoperative endocrinological deficit whenever the pituitary complex is involved (**Level C**).

### 4.2 Radiological assessment

*The EANS task force recommends that all patients with a newly discovered lesion compatible with a CSM undergo cerebral MRI with* 3D T1 post-gadolinium sequences, 3D T2 anatomical sequences, time-of-flight (TOF) angiographic sequences and Fat sat sequences *to assess the lateral/upward/posterior extension of the tumour in the parasellar area, the involvement of CNs II-VI, the overall anatomy of the region and the vasculature, in particular the cavernous segment of the ICA. A cerebral CT scan should also be performed to assess the presence of hyperostosis in the parasellar area when surgery is indicated. The hyperostosis can be seen with sufficient accuracy in T2-weighted images, whenever a CT scan cannot be performed (whatever the reason). As part of the preoperative planning, digital subtraction angiography (DSA) with balloon occlusion test to evaluate the ICA patency as well as tolerance for ICA occlusion can be undertaken (****Level C****).*

### 4.3 Patient counselling

The EANS task force recommends patient counselling prior to the treatment of a CSM in order to extensively discuss the risk and benefits of any surgical or non-surgical treatments and natural history of the disease, especially if asymptomatic. Perspectives in terms of QoL, functional impairment and mortality should also be openly discussed (**Level C**).

### 4.4 Conservative management

The EANS task force recommends that conservative treatment with serial imaging follow-up should be proposed in patients with a newly diagnosed asymptomatic CSM that has no mass effect on the adjacent temporal lobe (**Level C**). Whenever the CSM is suspected to be progestin-induced, hormonal treatment should be discontinued at first.

### 4.5 Radiation therapy and stereotactic radiosurgery

The EANS task force recommends that SRS or SRT (either single-dose or fractionated) should be considered in the following cases, insofar as the distance to the ON is superior to 3 mm (**Level C**):

- Asymptomatic, > 40 years old patients with a purely intracavernous CSMs <2.5 cm showing growth on serial imaging after initial conservative treatment;
- Asymptomatic patients with partly extracavernous CSMs showing growth on serial imaging after initial conservative treatment;
- Symptomatic patients with CSMs <2.5 cm, provided that the symptoms are not related to ON compression
- Symptomatic patients with partly extracavernous CSMs in whom surgery is contraindicated.
The EANS task force recommends that fractionated RT should be considered in cases that warrant treatment (see above) if the distance to the ON is less than 3 mm and the ipsilateral visual function is good (**Level C**).

## 5 Surgery

### 5.1 The EANS task force recommends proceeding to surgery in the following cases (**level C**)

- *Atypical lesions or unclear diagnosis*
- *Rapidly symptomatic lesions or unusual radiology*
- *Symptomatic CSMs*
- *Recurrence after radiation*
- *Aggressive tumour histology or behaviour*
- *Asymptomatic patients with growth of the extracavernous portion of the tumours on serial imaging*
- *Young patients (< 40 years) with asymptomatic, but growing CSM might be considered for surgery, if patient agrees.*
- *progressive visual loss due to ON compression*

### 5.2 Adjuvant therapy

The EANS task force recommends considering adjuvant SRS or f-SRT after subtotal surgical resections (**Level C**) whenever growth of residual tumour is observed during follow-up through tumour remnant volume analysis.

### 5.3 Follow-up

Patients diagnosed with CSMs should undergo an appropriate follow-up, including oncological, ophthalmological, endocrinological, neurological and neurosurgical assessment, and according to the latest EANO guidelines (Level C).

## 6 Conclusions

The initial evaluation of patients with a suspected CSM must include a clinical, ophthalmological, endocrinological and radiological assessment. Whenever a CSM is diagnosed, a thorough evaluation by a multidisciplinary team involving neurosurgeons, radiation oncologists, radiologists, ophthalmologists and endocrinologists is mandatory. Whatever the treatment chosen, the patients should be managed in tertiary referral centres.

Since surgical techniques evolved dramatically over the past twenty years, microsurgery should not be banned from the therapeutic armamentarium of CSMs, especially when it comes to aggressive lesions in young patients presenting with oculomotor, visual or endocrinological impairment (alternative: trigeminal dysfunction/neuralgia).

Should surgery be the first-line treatment decided, open cranial procedures seem to offer best tumour control and higher rates of GTR than the EEA, in particular when the tumour extends laterally to the lateral wall of the CS. Through the EEAs, a safe strategy of bony skull base decompression and limited tumour removal in the exophytic component of the tumour, outside the cavernous sinus, can be effective in most patients for alleviating symptoms and achieving tumour control when combined with RT. Surgery should not be advocated as first line treatment in small/asymptomatic lesions/in elderly patients. Both SRT and SRS offer excellent tumour control with low rates of oculomotor/visual complications. However, the mid-to – long-term risk of pituitary dysfunction is non-negligible.

## Compliance with ethical standards

### Funding

No funding was received for this research.

### Ethical approval

This article does not contain any studies with human participants performed by any of the authors.

## Authorship statement

The article was initiated by TRM, who had the original idea. MVC and TRM performed the literature search and the literature analysis. MVC and TRM drafted the article. TRM contacted the co-authors. All the co-authors critically revised the article and gave a substantial contribution in the improvement of the content of the manuscript.

## Declaration of competing interest

The authors declare that they have no known competing financial interests or personal relationships that could have appeared to influence the work reported in this paper.
